# Valeric Acid, a Gut Microbiota Product, Penetrates to the Eye and Lowers Intraocular Pressure in Rats

**DOI:** 10.3390/nu12020387

**Published:** 2020-01-31

**Authors:** Janusz Skrzypecki, Karolina Niewęgłowska, Emilia Samborowska

**Affiliations:** 1 Department of Experimental Physiology and Pathophysiology, Laboratory of Centre for Preclinical Research, Medical University of Warsaw, Banacha 1B, 02-097 Warsaw, Poland, nieweglowska.karolina@gmail.com; 2 Mass Spectrometry Laboratory, Institute of Biochemistry and Biophysics, Polish Academy of Sciences, 02-106 Warsaw, Poland; emi.sambor@gmail.com

**Keywords:** valeric acid, gut bacteria, intraocular pressure, blood pressure

## Abstract

Mechanisms controlling intraocular pressure (IOP) and arterial blood pressure (BP) share similar mediators, including gut bacteria metabolites. Here, we investigated the effects of valeric acid (VA), a short chain fatty acid produced by microbiota from undigested carbohydrates, on IOP and BP. To test if gut VA penetrates to the eye we evaluated eyes’ homogenates after the administration of D9-VA into the colon. Additionally, the following experimental series were performed on 16-week-old Sprague Dawley rats to analyze the influence of VA on IOP: vehicle treatment; VA treatment; VA + hydroxybutyrate - a short chain fatty acids’ G protein-coupled receptor 41/43 (GPR 41/43) blocker (ANT); hydroxybutyrate; VA + angiotensin II; angiotensin II; VA treatment in rats with superior cervical ganglion excision and sham operated rats. D9-VA rapidly penetrated from the colon to the eye. VA significantly decreased IOP and BP. The decrease in IOP was gradual and lasted through the experiment. In contrast, a decrease in BP was instantaneous and lasted no longer than 10 min. Angiotensin II, ANT, and sympathetic denervation did not influence the effect of VA on IOP. In conclusion, colon-derived VA penetrates to the eye and decreases IOP. The effect is independent from BP changes, angiotensin II, GPR41/43, and sympathetic eye innervation.

## 1. Introduction

The interplay between gut microbiota products and mechanisms regulating homeostasis is being investigated in many fields of medicine [1,2,3]. This complex relationship is believed to play an important role in autoimmune diseases, e.g., multiple sclerosis and inflammatory bowel disease, and in life-style diseases such as hypertension and atherosclerosis [1].

Short chain fatty acids (SCFAs), including butyric and valeric acids (VA), are produced by gut bacteria from undigested carbohydrates [4]. It is believed that they mediate beneficial cardiovascular effects of dietary products rich in oligosaccharides, including vegetables [5]. On the molecular level, it is suggested that SCFAs modulate blood pressure (BP) regulation via G-protein coupled receptors (GPCR), directly inhibit the intrarenal renin–angiotensin system [6], and interfere with the sympathetic nervous system [7]. Furthermore, recent data show that SCFAs might also mediate beneficial effects of dietary fiber, i.e., protection against cardiovascular disease, colorectal cancer, obesity, and diabetes [1].

Interestingly, although bacteria metabolites, including SCFAs, are well known players in BP research [8], the role of these compounds in intraocular pressure (IOP) regulation is obscure. However, considering that epidemiological and experimental data suggest that BP and IOP are under the influence of common neurohumoral regulatory mechanisms, i.e., sympathetic nervous system or the renin–angiotensin system, we hypothesize that SCFAs might have an effect on IOP as well [9]. Indeed, we have recently found that butyric acid lowers IOP in normotensive rats (WKY) [10]. Furthermore, previous findings suggest that hydrogen sulfide, a gut bacteria metabolite, might play a role in regulation of BP and IOP as well [11,12].

Here, we investigate the effect of valeric acid on the IOP and mechanisms that may underlie such an interaction.

## 2. Materials and Methods

The study was conducted according to the ARVO Statement for the Use of Animals in Ophthalmic and Vision Research, the EU Directive 2010/63/EU, and approved by the local bioethical committee.

Experiments were performed on 70 males, 12–16-week-old Sprague Dawley (SPRD) rats. Rats were delivered by the Central Animal Laboratory at the Medical University of Warsaw. Rats were housed in groups of 3, with access to water and chow ad libitum. A 12h-light/12h-dark cycle, temperature of 22 °C, and humidity of 45%–55% was maintained throughout the experiment.

All rats underwent placement of catheters into the femoral artery and femoral vein as described elsewhere [10]. VA (Sigma-Aldrich, Poznan, Poland) for all injections was prepared at a dose of 30 mg/kg. VA for a bolus injection was soluted in 1ml of PBS to ensure a stable pH of 7.4. All bolus injections of VA were performed via a Foley catheter (10 F) introduced into the colon. To ensure placement of the catheter into the colon and not into the rectum, the tip of the catheter was placed 8 cm from the anus. Intravenous injections were performed via a catheter placed into the femoral vein.

### 2.1. Distribution of VA to the Eye

To test the distribution of the VA to the eye, we administered a radioactive D9-VA at a dose of 30mg/kg via a Foley catheter introduced into the colon (*n* = 6). D9-VA was soluted in 1ml of PBH to ensure a stable pH of 7.4. Five minutes after infusion, rats were decapitated and whole eyeballs were collected for analysis. Homogenates of the eyeballs were prepared with 10% ethanol and stored in −80 °C for analysis. The analysis of D9-VA was performed using an Ultra Performance Liquid Chromatograph with a triple quadrupole mass spectrometer. The spectrometer was operated in multiple-reaction monitoring mode. Analysis was performed in a negative electrospray ionization mode and analytes were separated using a Waters BEH C18 column and Waters BEH C18 guard column. Mobile phase A consisted of 1 mL of formic acid in 1 L water, and mobile phase B consisted of 1mL of formic acid in acetonitrile. The flow rate of the mobile phase was set at 0.6 mL/min.

Sample preparation was performed as follows: 80 µL methanol (containing internal standards) was mixed with 40 µL of a sample. After vortexing, 20 µL of 3-Nitrophenylhydrazine hydrochloride (3NPH) solution was mixed with 20 µL of N-(3-dimethylaminopropyl)-N′-ethylcarbodiimide (EDC)-pyridine solution and the final solution was incubated at room temperature for 30 min. Subsequently, the solution was diluted to 1 mL with 15% aqueous acetonitrile, centrifuged, and an aliquot was injected into the apparatus.

Calibration points were prepared to define the relationship between the concentration and detector response for analytes. The calibration curve for D9-VA was generated by comparing a ratio of the peak area of the analyzed compound to the peak of the corresponding internal standard against known analyte concentrations. The limit of quantification (LOQ) for D9-VA was 0.1 µM. 

### 2.2. The Effect of Colon-administered VA on IOP and BP

BP was measured continuously via a catheter introduced into the femoral artery. IOP was measured as we previously described [10]. In short, a 30 G needle was inserted into anterior chamber of the eye. The needle was passed slightly anterior to the limbus. Both IOP and BP signals were received by a pressure transducer connected to the BIOPAC system and analyzed by Acq Software (Biopac Systems, Goleta, USA). 

### 2.3. Evaluation of the Mechanism Underlying the Effects of VA on IOP and BP

To evaluate if GPR41/43 receptors are involved in the effects of VA on IOP, injection of 30 mg/kg of beta-hydroxybutyric acid (an antagonist of GPCR) soluted in 1ml of PBS to ensure a stable pH of 7.4 was administered via a catheter placed into a femoral vein before treatment with VA (*n* = 8). To evaluate if the sympathetic nervous system is involved in the effects of VA on IOP, VA was administered in rats with superior cervical ganglion excision (*n* = 8) and sham operated rats (*n* = 8). Firstly, a cervical midline incision was performed ventrally and following dissection of connective tissue, a common carotid artery was visualized. The superior cervical ganglion was identified dorsally to the division of the common carotid artery into the internal and external carotid arteries and excised. In order to study whether VA might interfere with systemic and ocular effects of angiotensin II, we infused angiotensin II (40 ng/kg) intravenously via a catheter placed into the femoral vein. Bolus injection was performed simultaneously with intracolonic injection of either VA (*n* = 8) or 0.9% NaCl (*n* = 8). All experiments and measurements were performed under general anesthesia with urethane at a dose 1.5 g/kg^−1^ b.w.

### 2.4. Statistical Analysis

Data were tested for normal distribution with the Kolmogorov–Smirnov test. Differences between the groups were analyzed with two-way ANOVA for repeated measurements (R-MANOVA). Significance of the effect over time was tested with one-way ANOVA for repeated measurements. A *p* < 0.05 was set as the level of statistical significance. Statistical analysis was performed with Graphpad Prism (USA). 

## 3. Results

### 3.1. Distribution of VA into the Eye

**Figure 1:**
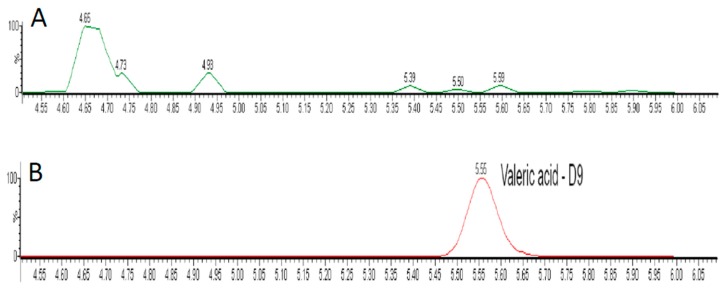
Chromatograms for valeric acid-D9 of the blank sample (**A**) and eye homogenate sample (**B**).

D9-VA was found in the homogenized eyeballs collected 5 min following colonic injection (Figure 1).

### 3.2. The Effect of Colon-derived Valeric Acid on IOP, BP, and HR

**Figure 2:**
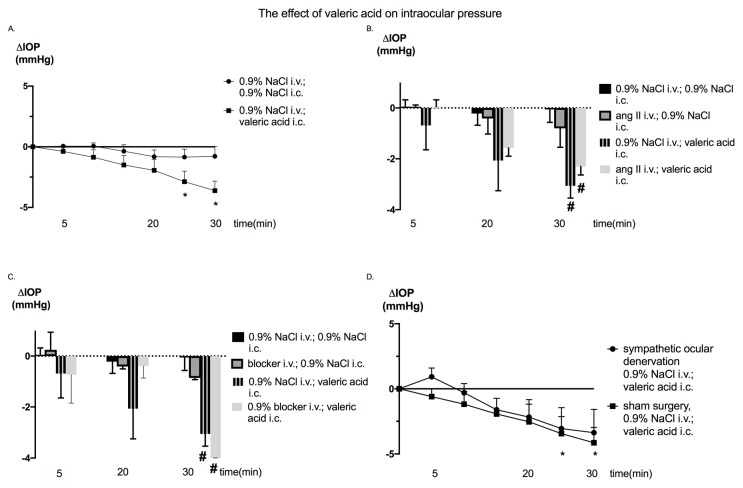
Changes in intraocular pressure (ΔIOP) in Sprague Dawley rats in response to the (**A**) intracolonic administration of valeric acid or 0.9% NaCl (vehicle), (**B**) intracolonic administration of valeric acid and intravenous injection of angiotensin II, (**C**) intracolonic administration of valeric acid and intravenous injection of the blocker (beta-hydroxybutyrate); injection: simultaneous intracolonic and intravenous injection of analyzed compounds, and (**D**) intracolonic administration of valeric acid and superior cervical ganglion excision; i.v.: intravenous injection; i.c.: intracolonic injection; * *p* < 0.05 vs. baseline, # *p* < 0.05 vs. 0.9% NaCl i.v.; 0.9% NaCl i.c.

**Figure 3:**
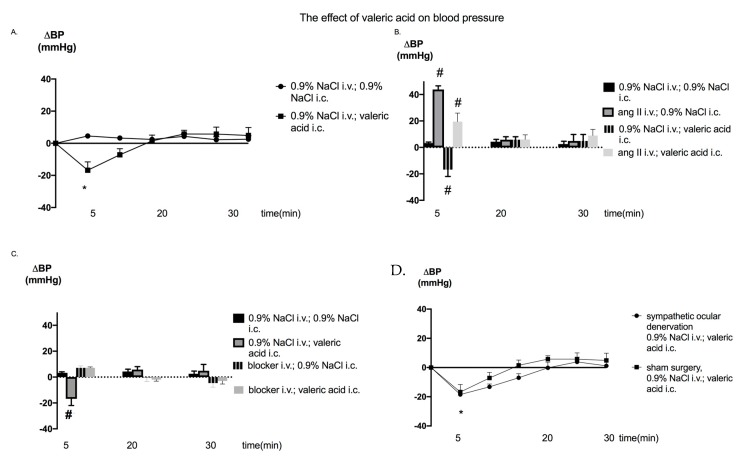
Changes in blood pressure (ΔBP) in Sprague Dawley rats in response to the (**A**) intracolonic administration of valeric acid or 0.9% NaCl (vehicle), (**B**) intracolonic administration of valeric acid and intravenous injection of angiotensin II, (**C**) intracolonic administration of valeric acid and intravenous injection of the blocker (beta-hydroxybutyrate); injection: simultaneous intracolonic and intravenous injection of analyzed compounds, and (**D**) intracolonic administration of valeric acid and superior cervical ganglion excision; i.v.: intravenous injection, i.c.: intracolonic injection; * *p* < 0.05 vs. baseline, # *p* < 0.05 vs. 0.9% NaCl i.v.; 0.9% NaCl i.c.

**Figure 4:**
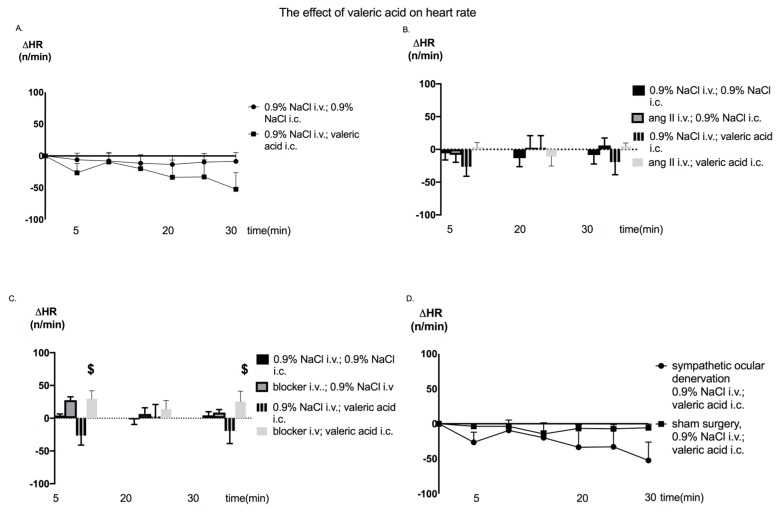
Changes in heart rate (ΔHR) in Sprague Dawley rats in response to the (**A**) intracolonic administration of valeric acid or 0.9% NaCl (vehicle), (**B**) intracolonic administration of valeric acid and intravenous injection of angiotensin II, (**C**) intracolonic administration of valeric acid and intravenous injection of the blocker (beta-hydroxybutyrate); injection: simultaneous intracolonic and intravenous injection of analyzed compounds, and (**D**) intracolonic administration of valeric acid and superior cervical ganglion excision; i.v.: intravenous injection, i.c.: intracolonic injection; $ *p* < 0.05 blocker i.v.; valeric acid i.c. vs. 0.9% NaCl i.v.; valeric acid i.c.

At baseline there were no significant differences between groups (Table 1). Injection of VA significantly lowered IOP (F_(6,84)_ = 3.87; *p* < 0.01) (Figure 2A). The effect was most pronounced 20 min following injection (F_(1.281, 8.965)_ = 13.18, *p* < 0.05) (Figure 2A). VA transiently decreased BP (F_(6, 84) = 10.19)_; *p* < 0.0001) (Figure 3A). This effect lasted 5 min. Injection of VA did not significantly influence heart rate (HR) (Figure 4A).

### 3.3. The effect of SCFA antagonist, sympathetic nervous system, and angiotensin II on IOP, BP, and HR effects of VA

Effects of the SCFA antagonist injection, sympathetic denervation, and angiotensin II injection on IOP, BP, and HR are presented in Figure 2, Figure 3 and Figure 4. 

The effect of VA on IOP was not influenced by injection of angiotensin II and beta-hydroxybutyric acid (Figure 2B,C). However, both angiotensin II and beta-hydroxybutyric acid diminished the systemic hypotensive effect of VA (Figure 3B,C). Cervical ganglion excision did not influence the effect of VA on IOP or BP (Figure 2D and Figure 3D). 

Furthermore, neither injection of angiotensin II nor ocular sympathetic denervation influenced HR in the setting of VA injection (Figure 4B,D). HR following injection of VA was significantly lower in comparison to the effect of injection of beta-hydroxybutyric acid or beta-hydroxybutyric acid combined with VA (F_(12, 126)_ = 2.142; *p* < 0.05) (Figure 4C).

## 4. Discussion

A novel finding of our study is that VA, a gut bacteria product, rapidly penetrates from the gut to the eye and decreases IOP. Gut-bacteria metabolites were found to affect numerous homeostatic processes including blood pressure or immune response [1]. We have recently shown that butyric acid might decrease IOP in normotensive rats, independently from changes in BP [10]. Here, we aimed to establish distribution of VA to the eye and its effects on IOP. Therefore, we infused a radiolabeled D9-VA and analyzed its presence in the homogenized ocular tissues. D9-VA was found in the eye as soon as 5 min following intracolonic injection. Considering that a significant ocular hypotensive effect was not observed until 20 min after injection of VA, we hypothesize that VA might have affected production or outflow of aqueous humor.

Furthermore, we wanted to test whether ocular and systemic effects of VA depend on interactions with specific receptors, i.e., GPCR or olfactory receptors [6,13]. Notably, we found that although beta-hydroxybutyric acid, a GPCR antagonist [14], attenuated the systemic hypotensive effect of VA, it did not affect the ocular effects of VA. This discrepancy might be related to non-specific binding of VA in the eye or its interaction with olfactory receptors [15], which could not be analyzed experimentally as antagonists of olfactory receptors are not available commercially. Since SCFAs suppress the renin–angiotensin system and affect the sympathetic nervous system [7,16], we wanted to test whether injection of angiotensin II or sympathetic ocular denervation affects systemic and ocular responses to VA. Here, we found that concomitant injection of angiotensin II blunts the systemic response to VA, but does not affect its ocular hypotensive effect. Furthermore, sympathetic ocular denervation did not affect the systemic nor ocular response to VA. These observations further support our hypothesis that SCFAs might influence IOP directly, regardless of their effects on systemic BP or neurohumoral regulatory mechanisms.

There was no significant effect of VA injection on HR. However, there were statistically significant differences of HR changes between injection of VA (a non-significant decrease from baseline) and beta-hydroxybutyrate or beta-hydroxybutyrate combined with valeric acid (a non-significant increase from baseline). It might be hypothesized that the effect of SCFAs on BP depends primarily on their interference with the sympathetic nervous system. In contrast, considering that beta-hydroxybutyric acid and sympathetic ocular denervation did not affect the IOP-lowering effect of VA, we hypothesize that SCFAs lower IOP independently from the sympathetic nervous system. A limitation of our study is that it was performed under general urethane anesthesia. Although urethane is widely utilized in studies on physiological processes [17], we cannot exclude its potential interference with our particular experimental setting. On the other hand, general anesthesia allows stress-free measurements. Furthermore, we decided to measure IOP invasively. The impact of a 30G needle paracentesis with physiological processes is difficult to assess. 

## 5. Conclusions

Intracolonic injection of valeric acid penetrates to the eye and decreases intraocular pressure independently from BP, angiotensin II, GPCRs, or sympathetic innervation of the eye. 

## Figures and Tables

**Table 1:** Baseline parameters of studied groups.

	IOP (mmHg)	MABP (mmHg)	HR (bpm)
0.9% NaCl i.v; 0.9% NaCl i.c.	23 ± 2	95 ± 3	352 ± 10
0.9% NaCl i.v.; valeric acid i.c.	24 ± 3	102 ± 2	339 ± 9
Ang II i.v.; valeric acid i.c.	21 ± 2	91 ± 4	315 ± 15
Ang II i.v.; 0.9% NaCl i.c.	21 ± 3	98 ± 5	325 ± 20
Sympathetic denervation; 0.9% NaCl i.v.; valeric acid i.c.	25 ± 4	105 ± 3	324 ± 8
Sham surgery; 0.9% NaCl i.v.; valeric acid i.c.	22 ± 4	103±2	333 ± 15
Beta-hydroxybutyrate i.v.; 0.9% NaCl i.c.	20 ± 1	99 ± 3	317 ± 13
Beta-hydroxybutyrate i.v.; valeric acid i.c.	23 ± 3	103 ± 4	345 ± 14

Baseline parameters are expressed as mean ± standard error of the mean; IOP: intraocular pressure, MABP: mean arterial blood pressure; HR: heart rate; i.v.: intravenous injection; i.c.: intracolonic injection; number of rats in all groups: *n* = 8.
